# Borax as a Cross-Linking Agent in Pectin Films: Effects of Borax Concentration and Temperature on Film Properties

**DOI:** 10.1021/acsomega.5c12753

**Published:** 2026-06-18

**Authors:** Natalia V. G. Mendes, Luís F. Zitei-Baptista, Fabricio B. Ferreira, Fabio R. M. Batista, Delia R. Tapia-Blácido, Eduardo R. Triboni

**Affiliations:** † Department of Chemical Engineering, Nanotechnology and Process Engineering Laboratory, Lorena School of Engineering, University of São Paulo, 12602-810 Lorena, SP, Brazil; ‡ Department of Chemistry, Laboratory of Agroindustrial Biopolymers, Ribeirão Preto Faculty of Philosophy, Sciences, and Letters, University of São Paulo, 14040-901 Ribeirão Preto, SP, Brazil; § Department of Biotechnology, University of São Paulo, 12602-810 Lorena, SP, Brazil

## Abstract

The present study evaluates the effect of borax as a cross-linker at different concentrations (0%, 1.32%, 2.61%, and 3.87%—w/w) on the physicochemical properties of the films comprised of pectin, glycerol, and borax. The film-forming solutions were made by two distinct preparation methods: i. a heated system, at 85 °C, without stirring (thermal method, TM) and ii. a nonheated high-speed stirring system, 30,000 rpm (agitation method, AM). Films produced by both methods were compared to assess their structural, thermal, mechanical, and antimicrobial properties. Steady-state and real-time infrared spectroscopy (FTIR) indicated the formation of B–O–C moieties, with cross-linking efficiency favored under both higher pH and temperature. SEM images and mechanical analyses revealed that AM gave rise to markedly coherent and continuous films, whereas TM-derived films exhibited surface defects and breakpoints, particularly at higher borax concentrations. Thermal analyses (TGA/DTA) indicated that borax enhances the thermal stability of the films regardless of the preparation method. Increasing borax content, however, generally reduced tensile strength and elongation at break, suggesting a competitive balance between cross-linking and plasticization. The films did not display significant antibacterial effects, likely due to partial solubilization during diffusion-based assays. Overall, the results demonstrate that temperature and borax concentration in the film-forming solution play a key role toward pectin film properties.

## Graphical abstract

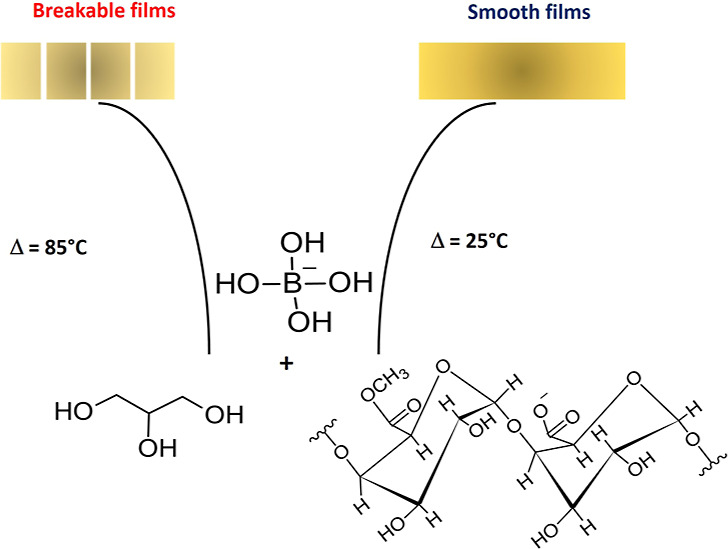

## 1 Introduction

In the midst of conventional plastics, biopolymer films emerge as alternative materials toward conventional plastics mainly in relation to food packaging technologies, offering notable advantages in sustainability and preservation.^1^ As a natural polysaccharide primarily extracted from citrus fruits and apples, pectin stands out as a very suitable raw material for biodegradable film development.^2^

Pectin films have high biocompatibility, biodegradability, and ability to form gas barriers.^3^ For all that, they can be suitable for a variety of applications including biocompatible materials for medical applications such as wound dressings and tissue regeneration matrices,^4^ ion transport channels,^5^ triboelectric nanogenerators,^6^ and optical sensing,^7^ coatings, and packaging for food storage/transport, and Bioplastic materials development.^8–13^

Pectin films, however, still present some drawbacks such as the poor mechanical strength and high sensitivity to moisture compared to synthetic polymers.^8,14^ The incorporation of plasticizers and cross-linking agents may help to overcome such limitations, providing hydrophobicity, mechanical integrity, and thermal resistance. Cross-linking requires agents with at least two reactive functional groups and results in more rigid structures with improved resistance to water, solvents, staining, and mechanical stress.^15^

Among cross-linking agents, borax and boric acids have drawn much attention, specifically for their effectiveness in forming network between chains nanocomposites and polysaccharide-based hydrogels.^16–20^ The cross-linking mechanism involves an equilibrium reaction between borax and water, converting borax into boric acid that undergoes hydrolysis to form the tetrahydroxyborate anion.^21–23^ This anion and other boron-based species can reversibly interact with hydroxyl groups giving rise to ester bonds, such as borate and boronate ester bonds.^17,20,24^ Bishop et al.^25^ found that the occurrence of boron-based cross-linking primarily occurs via preferential hydroxyl groups on the galactose side chains of guar gum or hydroxypropyl guar (HPG). Tetrahydroxyborate ions can also interact through hydrogen bonds with *cis*-hydroxyl groups of polymeric chains, as reported by Hurmaus and Plank.^26^

The efficiency of boron-based cross-linkers is usually increased by using polyols. Belcher, Tully, and Svehla^27^ provided early evidence that boric acid interacts with various polyols to generate complexes that strengthen acid–base reactivity. Legemah et al.^28^ produced novel boron cross-linkers with sorbitol, pentaerythritol, dipentaerythritol, N-glucosamine, and glyoxal.

Pectin films generally have poor antimicrobial activity which may restrict their applicability. By contrast, previous studies have shown the potential of boron compounds as antimicrobial agents.^29–33^ Gram-negative and Gram-positive bacteria have different cell wall structure.^34^ Gram-positive bacteria are known to have a thick and multilayered peptidoglycan wall, while Gram-negative bacteria have a thinner layer of peptidoglycans surrounded by an outer membrane of lipopolysaccharides. Comparing their resistance to antibiotics, Gram-positive bacteria are usually less resistant than Gram-negative bacteria.^34^

Although several studies have demonstrated the key role of boron compounds as cross-linkers in different systems, there is a lack of its specific influence on pectin films which requires further investigation toward the resulting material properties. Seong et al.^35^ studied pectin and tannic acid applied in PVA-borate hydrogels; Weizman et al.^36^ produced coating films composed of whey protein isolate (WPI), poly­(vinyl alcohol) (PVA), pectin, and borax; Ribeiro et al.^37^ filed a patent for antifungal coating films containing acetic acid, gelatin, pectin, propylene glycol, and borax; and Idahagbon et al.^38^ studied the application of borax in pectin–cellulose nanofiber composites for packaging applications.

We performed a systematic evaluation of how borax plays as a cross-linker agent in pectin films formed from casting of film-forming solutions comprised of glycerol, pectin, and borax. As pectin chain behavior is known to have pH and temperature dependency,^39–41^ two distinct preparative routes of the film-forming solutions that is thermal vs high-speed agitation are carried out in order to compare their influence on the structural, mechanical, physicochemical, and antimicrobial properties of pectin films. We have also considered how borax behaves in these different film-forming medium taking in account its content and reactivity with glycerol and pectin, hence, anticipating that temperature or stirring may provide specific bonds or interactions that may be correlated to the film outcomes. This survey assesses, therefore, the borax content, preparation method, and structure–property relationships in pectin-based materials, envisaging to given a better understanding of borax-mediated cross-linking mechanisms. The films were characterized by infrared spectroscopy (IR), thermogravimetry, differential thermal analysis (DTA), tensile tests, scanning electron microscopy (SEM), contact angle measurement, moisture content, and inhibition tests against *Escherichia coli* and *Bacillus subtilis* *.* SEM and mechanical analysis revealed that processing conditions critically determine network arrangement: agitation promotes uniform borate dispersion and spatially consistent cross-link density, while the thermal method leads to cracking films.

## 2 Methodology

### 2.1 Materials

Pectin GENU type USP/100, with 55–65% degree of esterification and 6.7–12% methoxy groups, was donated by CPkelco company. Glycerol was purchased by Synth, and borax (sodium tetraborate, 99.5–105%) was obtained from Nox Lab Solutions, and Milli-Q water.

### 2.2 Film-Forming Solution Preparation

Suspensions containing 1.0 g of pectin, 1.0 mL of glycerol (plasticizing agent), and 35 mL of Milli-Q water were prepared. Different concentrations of borax were added to the suspensions: ∼1.32% (PB1, 0.5 g of borax), ∼2.61% (PB2, 1.0 g of borax), and ∼3.87% (PB3, 1.5 g of borax) (%w/w, in relation to the total mass of the suspensions). A control film (P) without borax addition was also prepared. The samples were designated as shown in Table 1.

**Table 1:** Nomenclature of the Samples

sample	% borax (w/w)
P	0
PB1	1.32%
PB2	2.61%
PB4	3.87%

The suspensions were prepared by two different methods:

Method i: Pectin (1 g), glycerol (1 mL), and borax (at different concentrations) were previously mixed and heated at 85 °C for 2.5 h—thermal method (TM). Afterward, Milli-Q water (35 mL) was added and heated (85 °C) for 2.5 h to achieve dissolution.

Method ii: The suspensions were mixed and homogenized at 30,000 rpm until complete dissolution—agitation method (AM), as presented in Figure 1.

**Figure 1:**
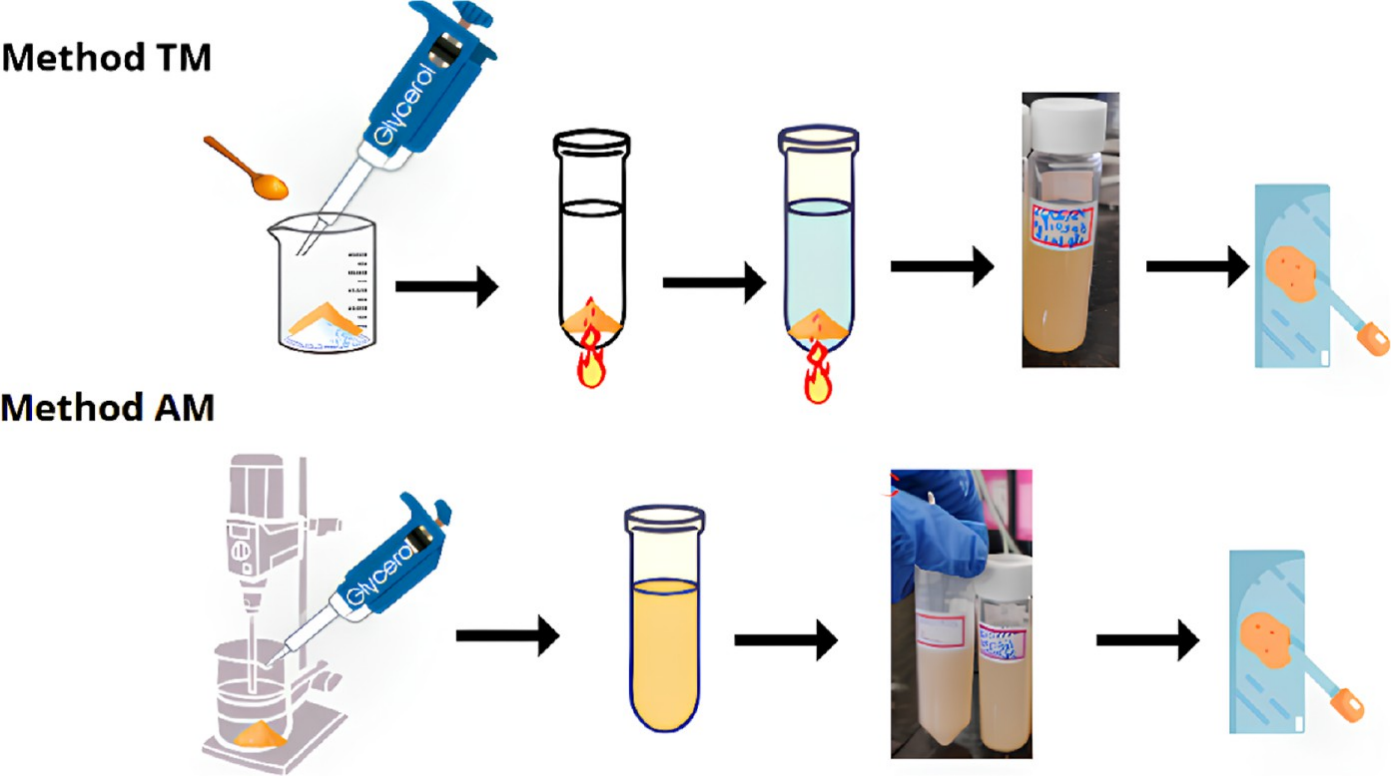
Methods of preparation of pectin-borax films. Method TM presented above and method AM shown below.

Finally, the film-forming solutions were deposited onto surfaces using a pipet and subsequently spread over the surfaces.

### 2.3 pH Measurement of the Film-Forming Solutions

The pH of the sample solutions (P, PB1, PB2, and PB4) was measured using the HI2550 HANNA Multiparameter pH/ORP/°C meter at 25 °C.

### 2.4 Infrared Measurements

ATR-FTIR analysis was used to determine functional groups and intermolecular interactions within films. The analyses were performed on a Cary 630 FTIR spectrometer (Agilent Technologies) equipped with a Diamond-ATR sampling module, with 32 scans over the wavenumber range of 4000–400 cm^–1^ and a resolution of 4 cm^–1^. The film-forming solutions were spread onto Teflon plates and left to dry at room temperature. Then, the resulting films were detached from the surface using spatulas and analyzed.

In order to understand the interactions between the species formed by borax hydrolysis and the hydroxyl groups of both pectin and glycerol, in situ IR spectroscopy (through infrared probe experiments–ReactIR 702L, Mettler Toledo) was carried out, monitoring the spectral pattern along with borax addition and at different temperatures in the systems composed of pectin (5 g)/water­(175 mL) and glycerol­(5 mL)/water­(175 mL). Spectral analysis was made by subtracting the background medium in order to bring out both the boron reactivity and interaction in each medium.

### 2.5 Scanning Electron Microscopy (SEM)

Scanning electron microscopy (SEM) (JSM-7401F, JEOL) imaged the surface morphology and homogeneity of the films. For SEM analysis, the film-forming solutions were deposited onto carbon tape and left to dry at room temperature and then coated with gold. Such a metallization of the samples was performed using the BAL-TEC MED 020 Coating System at an operating pressure of 10 psi, with a coating time of 25 s.

### 2.6 Thermogravimetry (TGA) and Differential Thermal Analysis (DTA)

Thermal analyses (TGA/DTA) were carried out to evaluate the thermal stability of pectin–borax films (PB 166/100, PB2, and PB4) in comparison with non-cross-linked pectin films (film P). A Netsch STA 449 F3 Jupiter thermal analyzer (Germany) was used, with an empty crucible as the reference. The heating rate was 10 °C/min, ranging from 25 to 500 °C, under an inert atmosphere. A sample mass of 10 mg was used for each test. Mass variations were recorded using a tungsten sensor, resulting in thermogravimetric curves (mass vs temperature) for each sample. From these data, decomposition points, thermal stability, and thermal events were identified.

### 2.7 Mechanical Properties’ Analysis

First, the film-forming solutions were spread and dried onto Teflon plates and then were detached from the plates to carry out the analysis. The mechanical properties of the film such as thickness and tensile strength were evaluated using tensile tests. Tensile strength and elongation at break were determined according to ASTM D882-95, using an average of five replicates for each case. The films were cut into strips measuring 25.4 mm × 130 mm using a scalpel and mounted between the grips of a TA.XT2i texture analyzer (SMS, Surrey, England). The initial grip separation was set to 80 mm, and the crosshead speed to 1.0 mm/s. Stress–strain curves were analyzed using Texture Expert V.1.15 software.

### 2.8 Antimicrobial Activity Analysis

Mueller–Hinton agar was used as the culture medium. Its composition includes beef extract, an acid hydrolysate of casein, starch, and agar, providing nutrients essential for bacterial growth. Starch acts as a protective colloid and energy source, while agar gives semisolid consistency. Two bacterial strains were selected: *B. subtilis* (Gram-positive) and *E. coli* (Gram-negative).

The bacteria were cultured in CASO broth and incubated at 37 °C for 18 h. After growth, the inocula were diluted in sterile saline to an optical density (OD) of 0.600 at 600 nm. Film discs (6 mm) were UV-sterilized (15 min on each side) to prevent microbial interference. Control tests were included. Controls were carried out to assess possible interferences in the analysis results.

Petri dishes containing Mueller–Hinton agar were inoculated with *B. subtilis* or *E. coli* using sterile swabs. For each sample, two dishes were prepared—one per bacterium. On each plate, three discs of the same sample were applied, allowing inhibition zones to be measured in triplicate. The antimicrobial performance of the pectin–borax films was specifically evaluated against *E. coli* (Gram-negative) and *B. subtilis* (Gram-positive), enabling assessment of the antimicrobial spectrum of the samples.

## 3 Results and Discussion

### 3.1 pH Measurement of the Film-Forming Solutions

Table 2 shows the pH values of the film-forming solutions, which bring out an increasing trend, from pH 3.5 to 8.1, as the borax concentration increased from 0% (w/w) to 4% (w/w) regardless of the method of preparation TM and AM. This pH increase is attributed to the increasing amount of borax, which in water dissociates into boric acid and hydroxide anions (eq 2) and then produces tetrahydroxyborate anions in solution,^21^ as shown in eq 3.

$${\mathrm{Na}}_{2}B_{4}O_{7}\cdot 10H_{2}O_{\left(s\right)}+H_{2}O_{\left(l\right)}⇋2{{\mathrm{Na}}^{+}}_{\left(\mathrm{aq}\right)}+B_{4}{{O_{7}}^{2-}}_{\left(\mathrm{aq}\right)}$$ 1

$$B_{4}{O_{7}^{2-}}_{\left(\mathrm{aq}\right)}+7H_{2}O_{\left(l\right)}⇋4H_{3}{BO}_{3(\mathrm{aq})}+2{OH^{-}}_{\left(\mathrm{aq}\right)}$$ 2

$$H_{3}{BO}_{3(\mathrm{aq})}+{OH^{-}}_{\left(\mathrm{aq}\right)}⇋{B(OH{)}_{4^{-}}}_{\left(\mathrm{aq}\right)}$$ 3

**Table 2:** pH of the Film-Forming Solutions

sample	pH thermal method (TM)	pH agitation method (AM)
film-forming solution P (control)	3.71	3.6
film-forming solution PB1	6.92	7.55
film-forming solution PB2	7.62	7.76
film-forming solution PB4	8.09	8.1

### 3.2 ATR-FTIR Measurements

Figure 2 shows the control films (P) produced via methods TM and AM, depicting characteristic peaks that can be assigned to the functional groups of the pectin and glycerol: O–H (3300–3270 cm^–1^) and C–H stretching (2937–2877 cm^–1^), symmetric COOH or C=O (1741–1745 cm^–1^)^42,43^ and asymmetric COO^–^ stretching (1600–1640 cm^–1^),^42^ C–H deformation (1410 cm^–1^), C–O–H and C–O deformation (1300 cm^–1^), C–O–C stretching (1100 cm^–1^), and C–O stretching (1010 cm^–1^).

**Figure 2:**
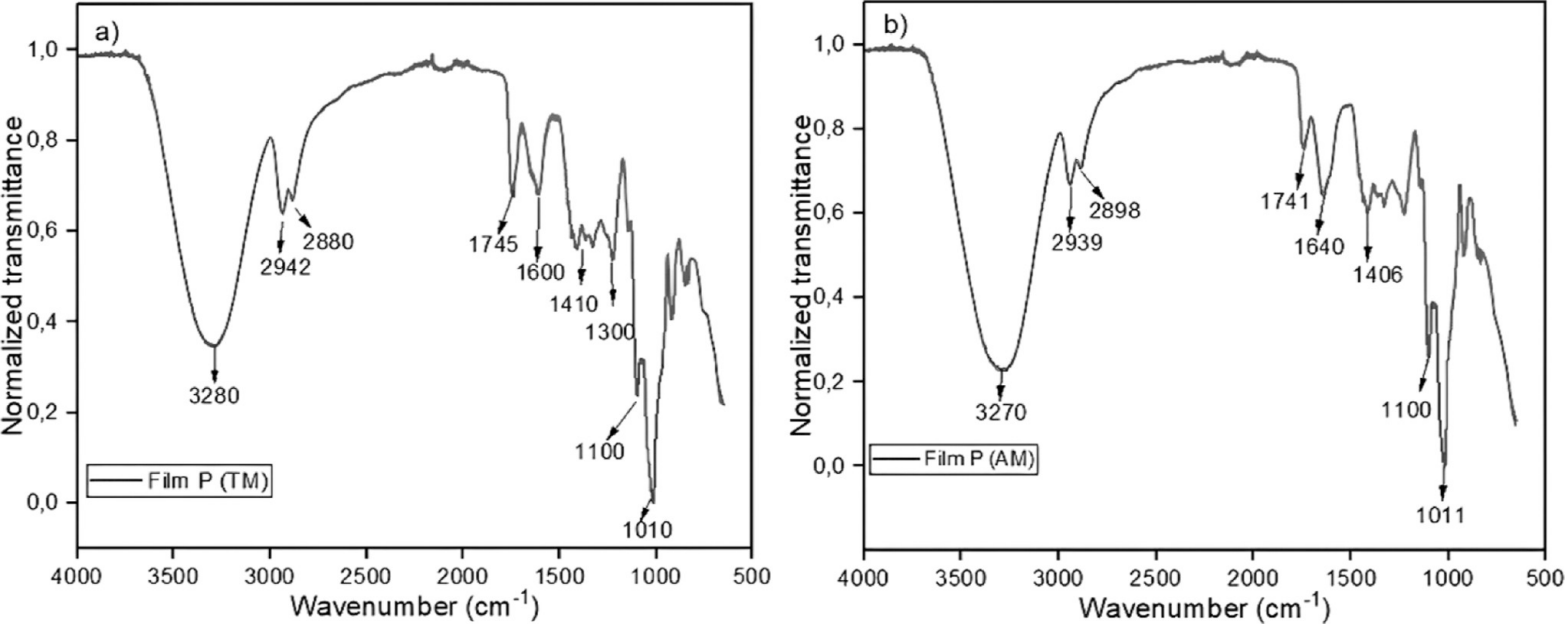
FTIR spectra of control samples prepared via: (a) method TM and (b) method AM.

Figures 3 and 4 show the ATR-FTIR spectra of the films prepared via TM and AM, respectively. In the films P (control) and PB1, there were evidence of vibrations associated with the C=O stretching (1748 cm^–1^), while the PB2 and PB4 films only showed COO^–^ (1618–1654 cm^–1^) and C–O (1085–1040 cm^–1^) stretches. The addition of borax led to the appearance of the band at ∼956 cm^–1^, which can be attributed to the B–O stretching of B­(OH)^−^_4_.^44,45^ Moreover, the peak of the O–H stretching in the range of 3100 cm^–1^ to 3400 cm^–1^ showed red shifts along with borax addition, which indicates the decrease of free hydroxyl groups. This result was also reported by Chen et al. and^46^ Lv et al.,^47^ and Thombare et al.^48^ mentioned the concumption of hydroxyl groups when borax was added in the presence of other polymers. Lv et al.^47^ observed a red shift of the O–H band from 3384 cm^–1^ to 3330 cm^–1^, while Chen et al.^46^ reported that from 3299 cm^–1^ to 3278 cm^–1^.

**Figure 3:**
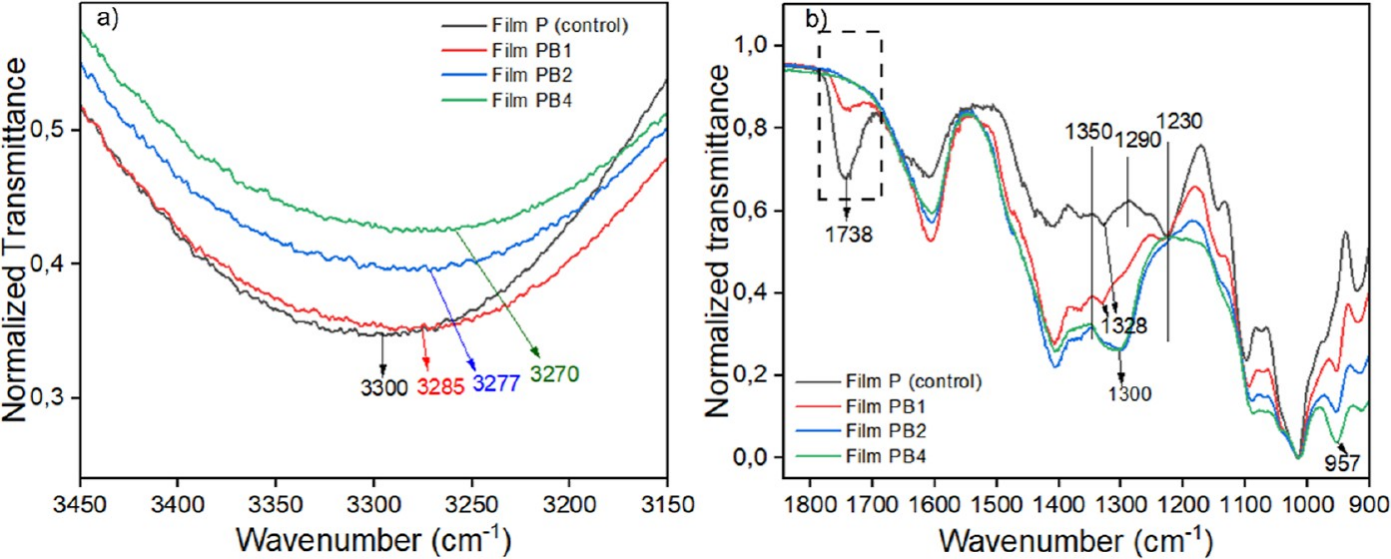
FTIR spectra of films prepared by the thermal method (TM): (a) from 3450 cm^–1^ to 3150 cm^–1^ and (b) from 1830 cm^–1^ to 900 cm^–1^.

**Figure 4:**
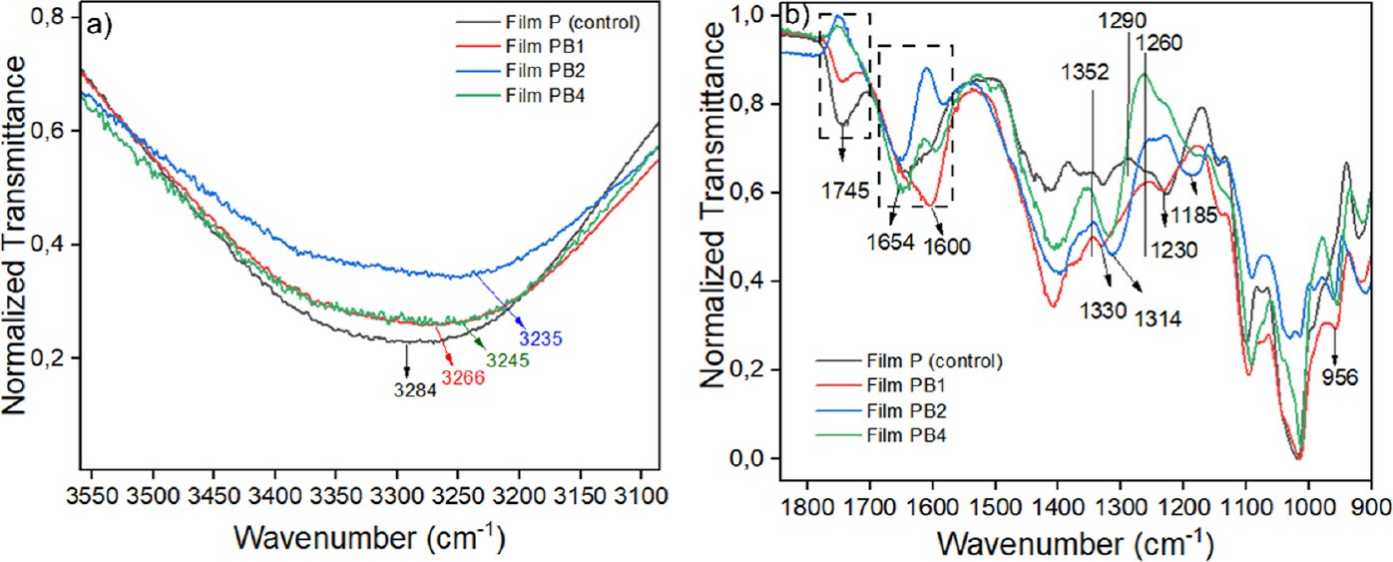
FTIR spectra of films prepared by the agitation method (AM) (agitated system route): (a) from 3560 cm^–1^ to 3050 cm^–1^ and (b) from 1830 cm^–1^ to 900 cm^–1^.

Broadening in the peaks between 1350/1330 and 1278 cm^–1^ can be attributed to the formation of borate ester, corresponding to the asymmetric B–O–C stretching and B–O vibrations.^46–52^ Moreover, the fading of the carbonyl stretching at both 1745 and 1738 cm^–1^ (HO–C=O) in the films with higher borax concentration (PB2 and PB4), indicates the occurrence of an acid–base reaction between pectin carboxyl and hydroxide groups, which are formed from borax hydrolysis.

Overall, these findings point out two main processes during film formation: i. an acid–base reaction of pectin carboxyl groups with the boron compound, generating carboxylate ions and ii. the interaction between hydroxyl groups and boron species and the formation of B–O–C bonds. However, hydrogen bonding between hydroxyl groups and boron species cannot be ruled out. Such reactivities or intermolecular interactions involving borate–diol moieties may explain the progressive network rearrangement observed in films with higher borax concentrations.

In order to address the activity of borax in the film-forming solutions in situ IR spectroscopy was performed. Spectral patterns of the film-forming solutions composed of glycerol/water at 50 °C and at room temperature are shown in Figure 5, whereas Figure 6 shows the spectral pattern of the solution composed of pectin/water at 50 °C and at room temperature. Control spectra of borax and boric acid in water at increasing concentrations are shown in Supporting Information, 1.

**Figure 5:**
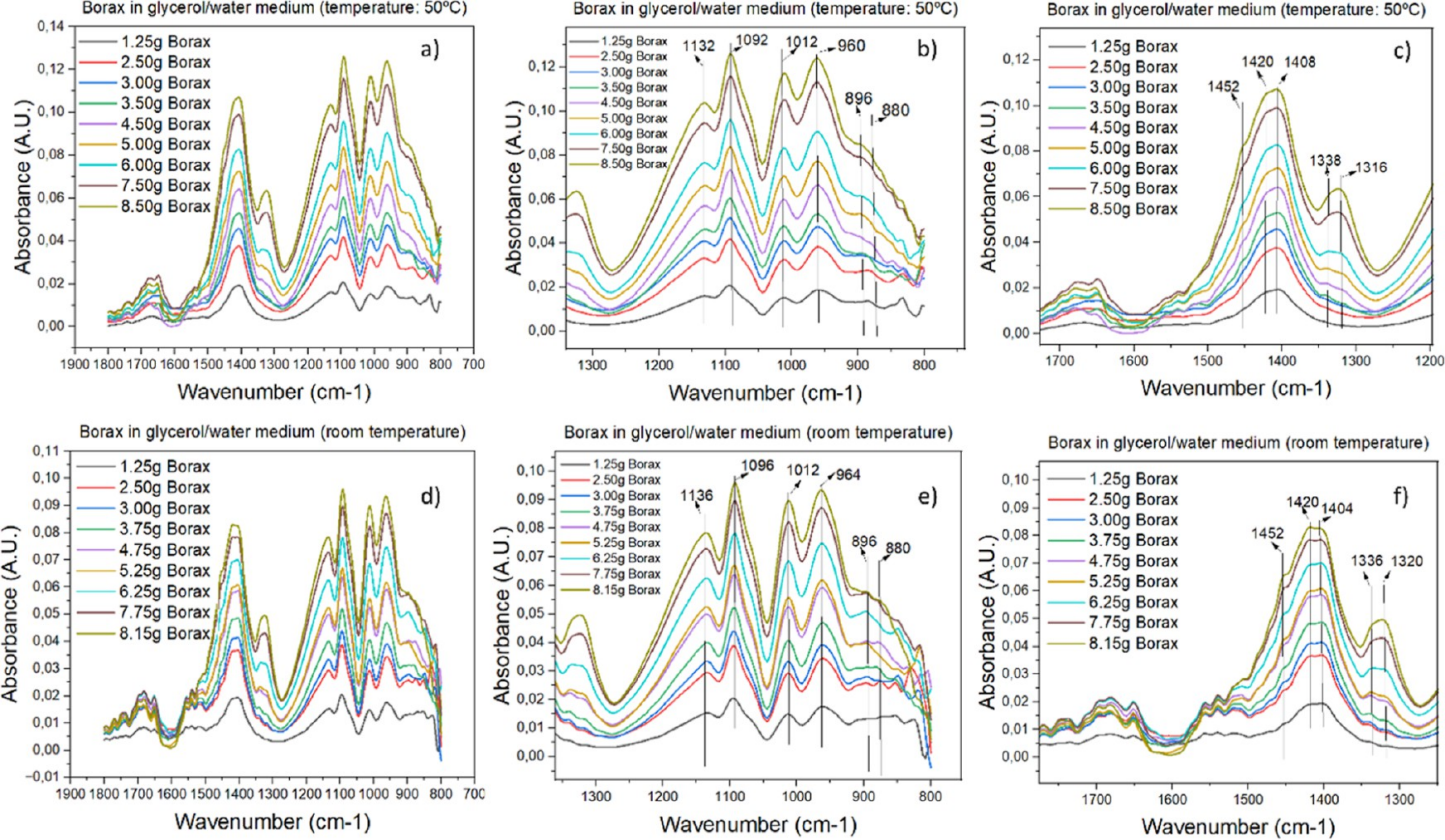
In situ spectroscopy analysis of borax in a glycerol (5 mL)/water­(175 mL) medium: at 50 °C (a–c) and at room temperature (d–f).

**Figure 6:**
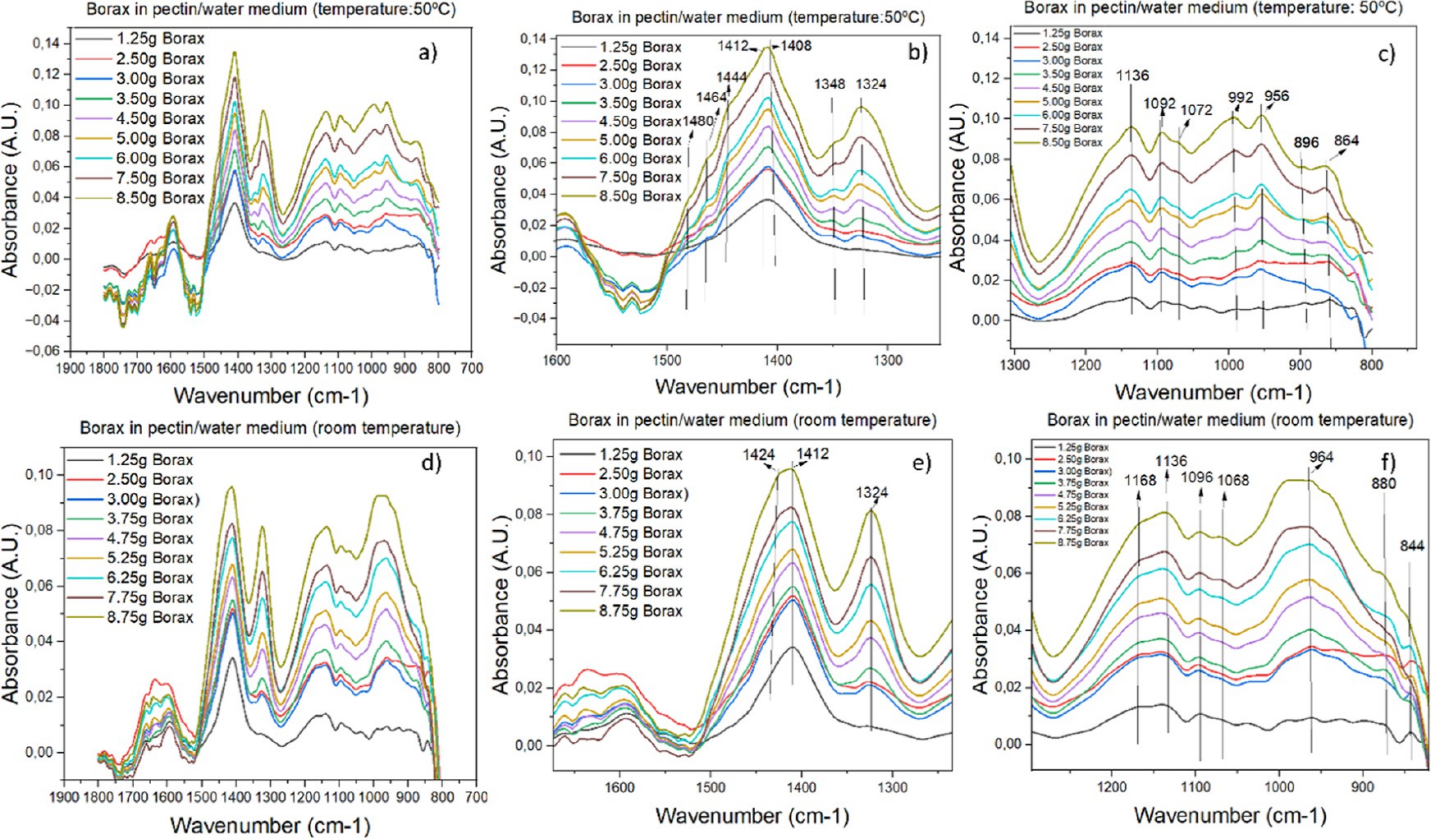
In situ spectroscopy analysis of borax in a pectin (5 g)/water (175 mL) medium: at 50 °C (a–c) and at room temperature (d–f).

Peaks in the region between 1140 cm^–1^ and 1000 cm^–1^ indicate modification of the stretch modes of the glycerol C–O and pectin C–O–C bonds as well as the B–O bond. The appearance of peaks in the region of 1108 cm^–1^ and 1140 cm^–1^ has been assigned to the formation of B–O–C.^53,54^ Therefore, along with the appearance of peaks in the range of 1330–1350 cm^–1^ and at 1420 cm^–1^, it seems that boron compounds and hydroxyl groups from both glycerol and pectin are most likely forming B–O–C bonds and hydrogen bonding as well (Figures 5 and 6), occurring in both trigonal and tetrahedral complexes.^55–57^ Moreover, pectin-borax IR spectra at room and at 50 °C in the region of 1320–1350 cm^–1^ presented different spectra behavior, suggesting that temperature affects the interactions/B–O–C bond formation, which may be related to pectin reactivity in different temperatures such as depolymerization.^39–41^ These events will be addressed in further studies.

#### 3.2.1 Scanning Electron Microscopy (SEM)

SEM analysis (Figures 7 and 8) revealed significant morphological differences among the samples depending on both the borax concentration and preparation method. In control samples (Figure 7) a dense structure was observed, which can be attributed to the higher number of hydrogen bonds between the molecules and the formation of B–O–C bonds. Structural changes induced by borax have also been reported for cellulose hydrogels by Tanpichai et al.^52^ Uyanga and Daoud^58^ also reported morphological changes caused by cross-linking agents in cellulose–chitosan systems.

**Figure 7:**
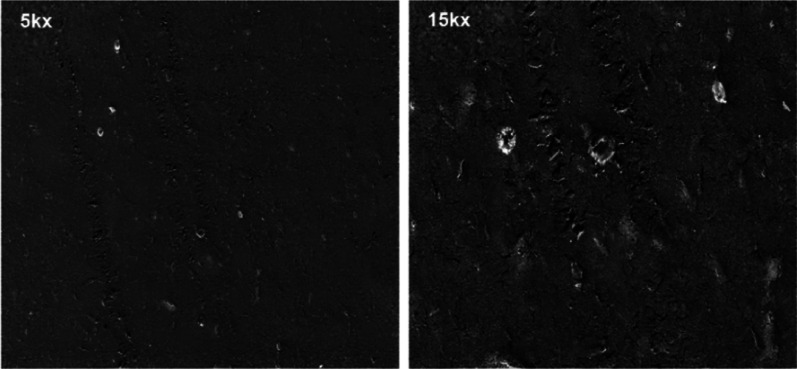
SEM micrographs of the control sample (non-cross-linked pectin film—Film P) at magnifications of 5000× (left) and 15,000× (right).

**Figure 8:**
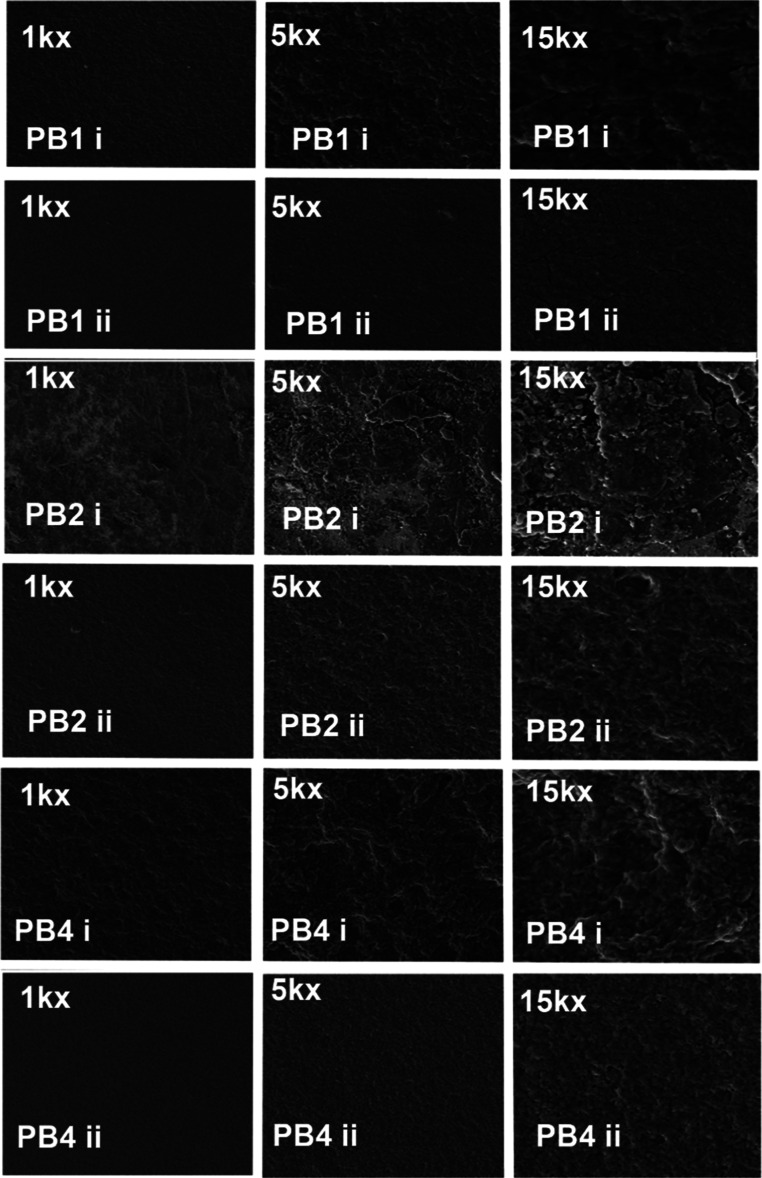
Scanning electron microscopy (SEM) of films prepared by methods TM (PB1 i, PB2 i, and PB4 i) and AM (PB1 ii, PB2 ii, and PB4 ii).

Upon borax addition, different trends are observed: TM prepared films presented rougher and more heterogeneous surfaces, resulting in porous and reliefs formation, especially at higher borax concentrations (PB2 and PB4). In contrast, films prepared using the agitation method (AM) showed smoother and more homogeneous surfaces compared to the thermal method (TM), as observed for PB1, suggesting that stirring promoted better dispersion. Therefore, the excess of borax (PB2 and PB4) under the thermal method seems to present internal stress leading to the occurrence of breakpoints during the drying process (as Figure 9). This feature might be attributed to depolymerization of the pectin chain under alkaline conditions (e.g., β-elimination–Supporting Information-2).^39–41^ Hence, such chemical stability of the pectin chain and the formation of boron-hydroxyl moieties may account for the favorable breakpoints in the films derived from heated film-forming solutions. Overall, SEM images clearly reveal an increase in surface roughness with increasing borax amount.

**Figure 9:**
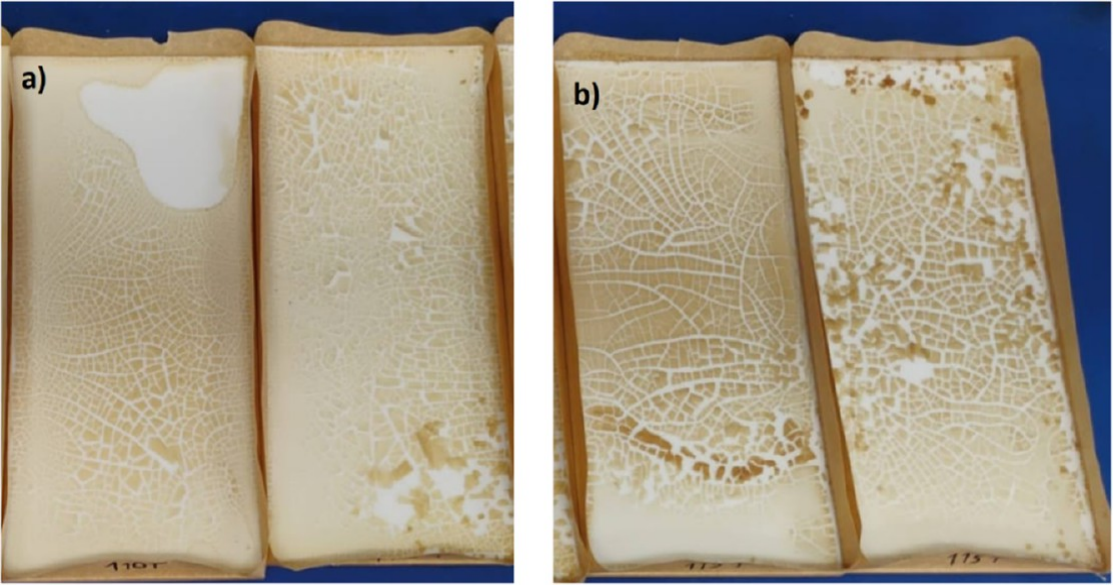
Breakpoints in films: (a) PB2 and (b) PB4 prepared by method TM.

### 3.3 Visual Aspect

As shown in Figure 10, all of the produced films exhibited a visually homogeneous appearance, were easy to detach from the Teflon plates, and presented transparency and a characteristic coloration associated with pectin.

**Figure 10:**
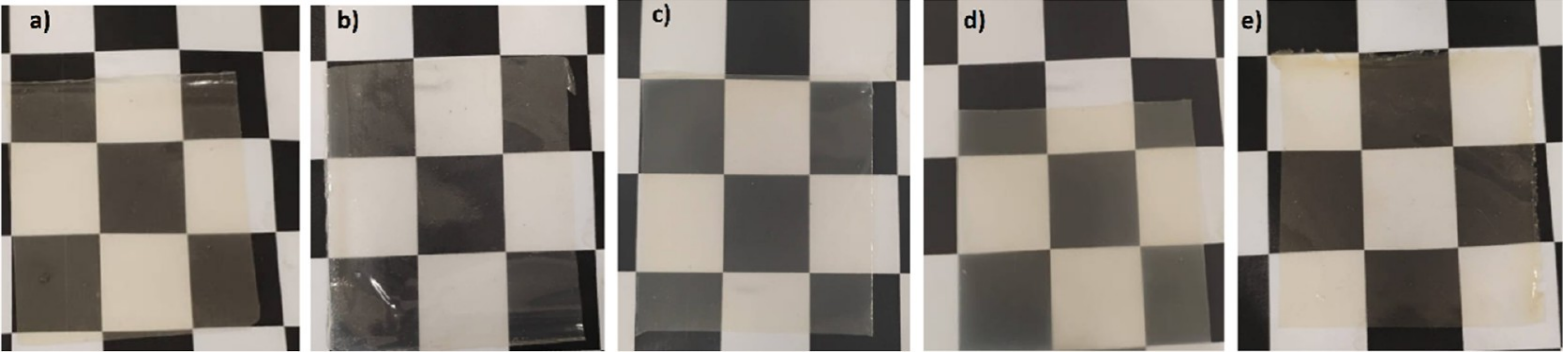
Photos of the films: (a) Film P – method TM; (b) Film P – method AM; (c) Film PB1 – method AM; (d) Film PB2 – method AM; and (e) Film PB4 – method AM.

### 3.4 Thermogravimetry and Differential Thermal Analysis (DTA)

TGA and DTA curves (Figures 11 and 12) showed that the method of preparation of film-forming solutions did not interfere in the thermal behavior of the films obtained, being mainly influenced by the concentration of borax. Initially, a dehydration stage was observed that increased proportionally with the addition of borax (Figure 11), as also reported by Pinto.^59^ Subsequently, the biopolymer matrix and glycerol degradation were observed followed by the final stage of decomposition.

**Figure 11:**
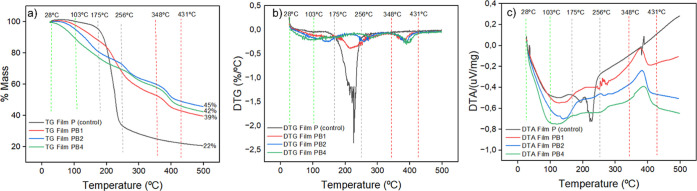
(a) TGA, (b) DTG, and (c) DTA of films prepared by method TM.

**Figure 12:**
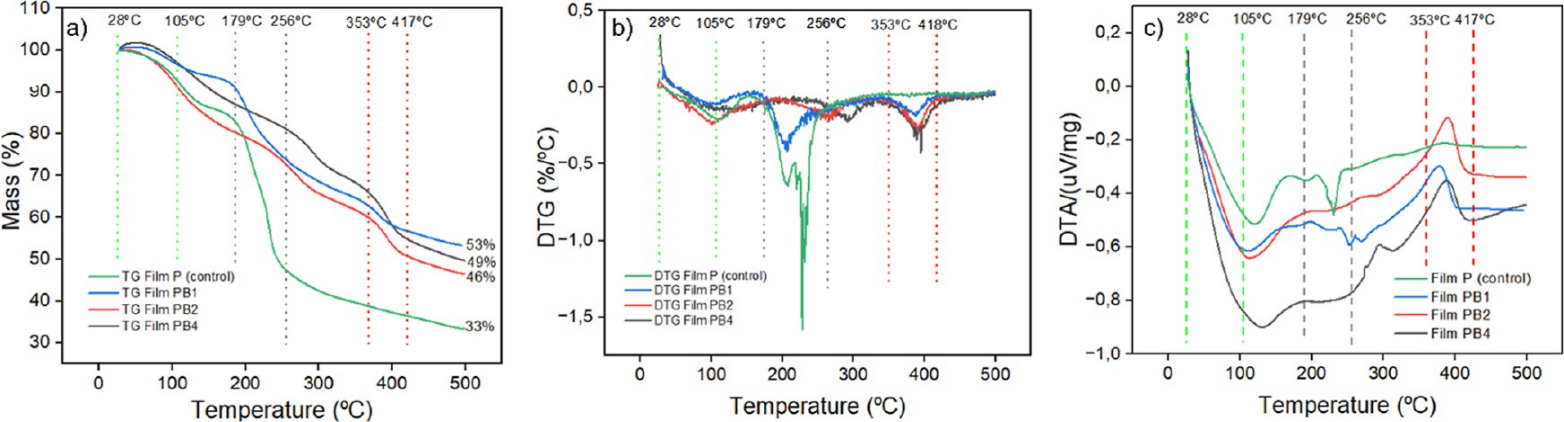
(a) TGA; (b) DTG; and (c) DTA of films prepared by method AM.

The analysis of the TGA and DTG curves revealed that films containing higher concentrations of borax (PB1, PB2, and PB4) exhibited greater mass loss between approximately 28 and 105 °C. This behavior can be attributed to dehydration, corresponding to an endothermic event, as shown in the DTA curve. Thus, the higher amount of borax the higher dehydration rate, leading to a greater mass variation in this temperature range, Figure 11a and b. Pinto^59^ described that the increase in borax content in geopolymer slurries resulted in higher mass losses within the range of 26–150 °C.

In the region between approximately 179 and 256 °C, the control sample presented an intense decomposition, characterized by a significant and rapid mass loss, while the films containing borax (PB1, PB2, and PB4) showed a more gradual mass loss over this interval. Hence, borax is contributing to a greater thermal stability in these films. Films with borax (PB1, PB2, and PB4) also showed another mass loss seen in the TGA and DTG curves between 350 and 417 °C. Tanpichai et al.^52^ and Uddin et al.^60^ related this step to the complexation between cellulose and boron in cellulose-based hydrogels and hybrid chitosan/cellulose nanofribils films, respectively.

### 3.5 Mechanical Properties and Contact Angle of Films

Mechanical properties of the P, PB1, PB2, and PB4 films prepared by method AM are shown in Tables 3 and 4, whereas by method TM, only the control sample (P) was analyzed because those films containing borax presented breakpoints. In addition, Table 3 presents the moisture content, and contact angle results of the control samples, showing a significant difference between the methods studied (TM and AM) (both presented in Section 2.2). Method AM yielded films with improved mechanical properties when compared to the method TM. In addition, the AM method yielded P films more humid than the TM method while tensile strength and elongation increased by ∼5 MPa and 5%, respectively. The Young’s modulus in method AM also showed a gain of ∼64 MPa. However, the contact angle decreased by ∼2.23°, indicating that the thermal method (TM) positively affected the functional properties of the films (moisture resistance and wettability).

**Table 3:** Comparison of Moisture Content, Mechanical Properties, and Contact Angle of Films Subjected to Different Methods (TM and AM)^a^

film	moisture (%)	thickness (mm)	tensile strength (MPa)	elongation at break (%)	Young’s modulus (MPa)	contact angle (°)
P (TM)	45.34 ± 0.79a	0.09 ± 0.00a	15.30 ± 0.69b	16.08 ± 1.61b	205 ± 34b	64.18 ± 0.14a
P (AM)	52.55 ± 0.31b	0.09 ± 0.06a	20.36 ± 0.18a	21.09 ± 0.36a	269 ± 6a	61.95 ± 0.08b

a Different lowercase letters in the same column indicate a significant difference between the types of film according to Tukey’s analysis (*p* < 0.05).

**Table 4:** Comparison of Moisture Content, Mechanical Properties, and Contact Angle of Films Treated with Different Borax Concentrations^a^

film	moisture (%)	thickness (mm)	tensile strength (MPa)	elongation at break (%)	Young's modulus (MPa)	contact angle (°)
P (AM)	52.55 ± 0.31a	0.09 ± 0.06c	20.36 ± 0.18a	21.09 ± 0.36a	269 ± 6a	61.95 ± 0.08a
PB1 (AM)	26.66 ± 0.64b	0.10 ± 0.01bc	9.50 ± 0.51b	12.52 ± 0.14c	141 ± 9b	31.93 ± 0.23c
PB2 (AM)	26.26 ± 0.85b	0.27 ± 0.02a	1.87 ± 0.12d	19.93 ± 0.32b	23 ± 2d	32.28 ± 0.07c
PB4 (AM)	22.68 ± 0.35c	0.21 ± 0.01b	2.32 ± 0.19c	6.67 ± 0.34d	69 ± 8c	38.39 ± 0.44b

a Different lowercase letters in the same column indicate a significant difference between the types of film according to Tukey’s analysis (*p* < 0.05).

Table 4 shows the moisture content, mechanical properties, and contact angle of films prepared by method AM with addition of borax.

Films with different borax concentrations using method AM showed a decrease in moisture content ranging from 52.55 ± 0.31 to 22.68 ± 0.35 (%), while the contact angle exhibited a significant drop of ∼30°from the control film to film PB1 (AM), followed by an increase from 31.93 ± 0.23 to 38.39 ± 0.44° with higher cross-linker additions. The film thickness also increased with the addition of the cross-linker, varying from 0.09 ± 0.06 to 0.27 ± 0.02 mm, with its maximum at film PB2 (AM). Tensile strength and YM displayed a similar pattern of substantial loss of these properties, ranging from 20.36 ± 0.18 to 1.87 ± 0.12 MPa for tensile strength and from 269 ± 6 to 23 ± 2 MPa for YM, with their minimum at sample PB2 (AM). The effect of borax addition also led to a decrease in film elongation, ranging from 21.09 ± 0.36 to 6.67 ± 0.34, with the lowest value in sample PB4 (AM).

Films produced by Nguyen et al.^61^ with pectin from two different fruit sources (Ambarella and Jackfruit) and cross-linked with different cations showed tensile strength and YM values ranging from ∼1 to 9 MPa and ∼18 to 309 MPa, respectively. However, Zhang et al.^62^ managed to produce pectin films with superior tensile strength and elongation properties using mango peel pectin.

The decrease in tensile strength and Young’s modulus in PB1 and PB2 can be assigned to the borax–glycerol–pectin system that undergoes competitive interactions and bonding, as shown in Section 3.2, hence, playing a key role on pectin film properties. Heated film-forming solutions seem to have larger contribution of glycerol-boron and pectin-boron covalent bonds than those prepared at room temperature, as a result, decreasing the efficiency of glycerol in acting as a plasticizer and then leading to less flexibility. Our assumption is based on studies addressed by Coer et al.^63^ involving glycerol and boric acid, where the more glycerol-boron bonds are formed, the less flexible the films were.

According to the Wenzel model,^64^ the topography of the surface can affect the contact angle; in this case, the roughness resulted in lower contact angles.^64,65^

Compared to the control sample, the decrease in tensile strength, elongation, and contact angle may also be connected to the material’s morphology. SEM micrographs (Figure 8) revealed an increase in the surface roughness of the films and more irregular surface with increasing borax concentration, which points out the formation of micro discontinuities in the polymer network, which may act as stress concentration points and reduce the material’s ability to deform and resist to rupture.

### 3.6 Antimicrobial Activity Analysis

The antimicrobial potential of the pectin–borax films was assessed against *E. coli* (Gram-negative) and *B. subtilis* (Gram-positive). However, while borax showed inhibition of fungal growth in solutions, antimicrobial tests against bacteria showed no clear inhibition halos for the films (P, PB1, PB2, PB4), as shown in Figure 13. Instead, partial dissolution of the film discs occurred upon contact with the agar medium, limiting their integrity during the assay. This dissolution likely released pectin, which may have served as a nutrient source for the bacteria, particularly for *B. subtilis*, which possesses enzymatic pathways for polysaccharide degradation. This phenomenon could mask any potential antibacterial effect and explains the increased bacterial density observed around the discs.

**Figure 13:**
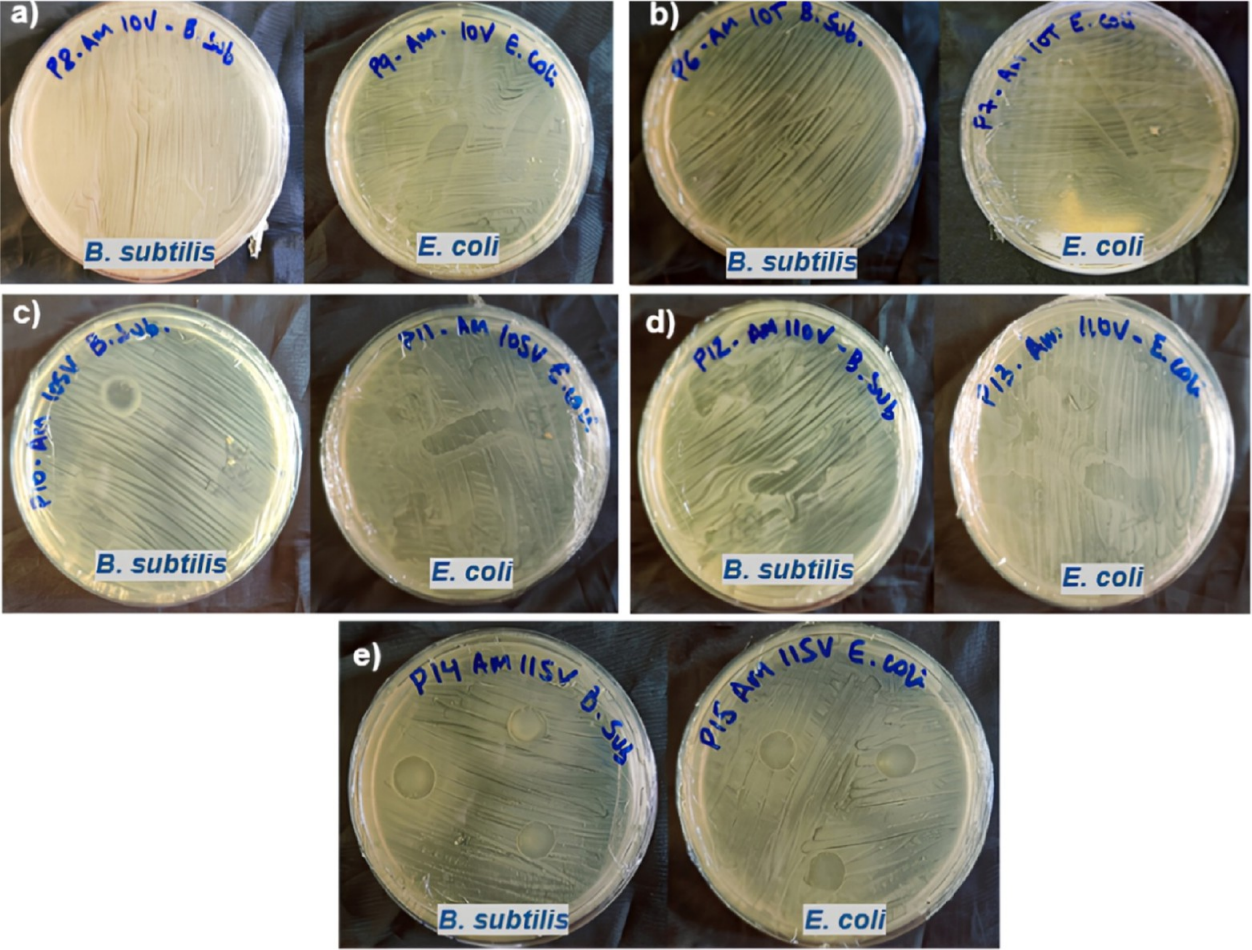
Antimicrobial effects of borax analyses in films: (a) P (control) prepared by method AM; (b) P (control) prepared by method TM; (c) PB1 by method AM; (d) PB2 by method AM; and (e) PB4 by method AM.

These results indicated that, under the tested conditions, borax did not confer a significant antibacterial functionality to the pectin films. However, several studies^66–68^ have shown that the incorporation of essential oils into biopolymeric matrices has resulted in satisfactory antimicrobial activity. Still, future studies are needed in order to explore alternative strategies to improve antibacterial performance, such as combining these films with known antimicrobial agents and reducing water solubility to prevent disc dissolution during assays.

## 4 Conclusions

We determine that both the borax concentration and the methods of preparation of the film-forming solutions play a key role in the morphology and mechanical and thermal properties of films made of pectin, glycerol, and borax. Borax dissociation in water forms tetrahydroxyborate anions that can interact or react with hydroxyl groups of the glycerol and pectin, giving rise to different kinds of boron–carbon moieties. Increasing the borax amount also increases the pH of the film-forming solutions, occasioning at least an acid–base reaction with pectin carboxyl groups. These molecular features seem to have a significant impact toward film properties.

The pectin films obtained from the thermal preparation had poor uniform textures showing three-dimensional clusters, whereas films from the agitation method, at the same borax concentration, presented better homogeneity without breakpoints, resulting in a better cohesion and adhesion onto surfaces. These findings may be attributed to the larger contribution of pectin-borax and glycerol-borax structures formed when using thermal preparation. Moreover, in general, increasing the borax amount gave rise to films with higher thermal stability and hydrophilicity but lower mechanical resistance and flexibility.

In contrast to previous studies reporting the antimicrobial effectiveness of boron compounds, the addition of borax to pectin-based films did not lead to significant antimicrobial inhibition.

However, borax reactivity and its speciation in solution are of significant influence on the properties of pectin-based films by interacting with available bearing-hydroxyl components, hence, it should be considered in the development of functional materials based on biopolymers and polyols that use borax as a cross-linker.

## Supplementary Material



## Notes

Real-Time Spectroscopy spectra of boric acid and borax in water medium at 50 °C, of pectin in water medium at 50 °C, and of boric acid in water/glycerol medium and in pectin/water medium and possible formation of B–O–C bonds (PDF)

## References

[1] Roy S., Priyadarshi R., Łopusiewicz Ł., Biswas D., Chandel V., Rhim J.-W. (2023). Recent Progress in Pectin Extraction, Characterization,
and Pectin-Based Films for Active Food Packaging Applications: A Review. Int. J. Biol. Macromol..

[2] Souza V. G. L., Mello I. P., Khalid O., Pires J. R. A., Rodrigues C., Alves M. M., Santos C., Fernando A. L., Coelhoso I. (2022). Strategies
to Improve the Barrier and Mechanical Properties of Pectin Films for
Food Packaging: Comparing Nanocomposites with BilayersCoatings. Coatings.

[3] de
Morais Lima M. (2024). FILMES BIODEGRADÁVEIS PARA EMBALAGENS DE ALIMENTOS:
AVANÇOS RUMO À PLENA SUSTENTABILIDADE. Revista Tópicos.

[4] Eivazzadeh-Keihan R., Noruzi E. B., Aliabadi H. A. M., Sheikhaleslami S., Akbarzadeh A. R., Hashemi S. M. (2022). Recent Advances on Biomedical
Applications of Pectin-Containing Biomaterials. Int. J. Biol. Macromol..

[5] Wang L. H., Kim T. H., Costanza V., Higdon N. J., Daraio C. (2023). Ion Transport
in Thermally Responsive Pectin Films. Appl. Phys. Lett. Applied Physics
Letters. Appl. Phys. Lett..

[6] Patnam H., Graham S. A., Manchi P., Vasant Paranjape M., Yu J. S. (2022). Eco-friendly pectin polymer film-based triboelectric nanogenerator
for energy scavenging. Nanoscale.

[7] Nazari S., Khiabani M. S., Mokarram R. R., Hamishehkar H., Chisti Y., Tizchang S. (2023). Optimized Formulation
of Polyaniline-Pectin
Optical Film Sensor for pH Measurement. Materials Science and Engineering
B: Advanced Functional Solid-State Materials. Mater. Sci. Eng., B.

[8] Espitia P. J. P., Du W.-X., Avena-Bustillos R., Soares N. F. F., McHugh T. H. (2014). Edible
Films from Pectin: Physical-Mechanical and Antimicrobial Properties
– A Review. Food Hydrocolloids.

[9] Suyatma N. E., Ishikawa Y., Kitazawa H. (2013). Nanoreinforcement
of Pectin Film
to Enhance its Functional Packaging Properties by Incorporating ZnO
Nanoparticle. Materials, Industrial, and Manufacturing Engineering
Research Advances. Adv. Mater. Res..

[10] Oliva-Moreno E. E., Encinas A. (2021). Addition of Pine Rosin
to Pectin Bioplastic films for
improved water resistance. Materials Letters. Mater. Lett..

[11] Souza V. G. L., Mello I. P., Khalid O., Pires J. R. A., Rodrigues C., Alves M. M. (2022). Strategies
to Improve the Barrier and Mechanical
Properties of Pectin Films for Food Packaging: Comparing Nanocomposites
with Bilayers. Coatings. Coatings.

[12] Guruchandran S., Prasath B. B. R., Sudhakar S., Mani E. (2024). Development
of Hematite
Nano Ellipsoids/Pectin Composite Films for Green Packaging Applications. Langmuir.

[13] Chaves M. L. C., Jesus G. A. M., Castro M. C., Bruni A. R. S., Monteiro J. P., Santos Junior O. O., Martins A. F., Bonafé E. G. (2025). Biodegradable
pectin/starch-based films applied on fresh pears. ACS Omega.

[14] Chambi H. N., Grosso C. R. (2011). Mechanical and water vapor permeability properties
of biodegradables films based on methylcellulose, glucomannan, pectin
and gelatin. Food Sci. Technol..

[15] Ollé L., Bacardit A., Morera J. M., Bartolí E., Argelich G. (2007). Cross-Linked Polymers for Aqueous
Finishing. Binders
Crosslinked with Polyaziridine. Part I: Behaviour of Polyurethane. J. Soc. Leather Technol. Chem..

[16] Tantiwatcharothai S., Prachayawarakorn J. (2020). Property improvement of antibacterial wound dressing
from basil seed (O. basilicum L.) mucilage- ZnO nanocomposite by borax
crosslinking. Carbohydr. Polym..

[17] Aeridou E., Diaz Diaz D., Alemán C., Pérez-Madrigal M. M. (2020). Advanced
Functional Hydrogel Biomaterials Based on Dynamic B–O Bonds
and Polysaccharide Building Blocks. Biomacromolecules.

[18] Seidi F., Jin Y., Han J., Saeb M. R., Akbari A., Hosseini S. H. (2020). Self-Healing
Polyol/Borax Hydrogels: Fabrications, Properties and
Applications. Chem. Rec..

[19] Liu C., Lei F., Li P., Wang K., Jiang J. (2021). A Review on Preparations,
Properties, and Applications of Cis-Ortho-Hydroxyl Polysaccharides
Hydrogels Crosslinked with Borax. Int. J. Biol.
Macromol..

[20] Palungan J., Luthfiyah W., Mustopa A. Z., Nurfatwa M., Rahman L., Yulianty R. (2024). The Formulation and Characterization of Wound
Dressing Releasing S-Nitrosoglutathione from Polyvinyl Alcohol/Borax
Reinforced Carboxymethyl Chitosan Self-Healing Hydrogel. Pharmiaaceutics.

[21] Nianyin L., Yu J., Daocheng W., Chao W., Jia K., Pingli L., Chengzhi H., Ying X. (2022). Development status of crosslinking
agent in high-temperature and pressure fracturing fluid: a review. J. Nat. Gas Sci. Eng..

[22] Mesmer R. E., Baes C. F., Sweeton F. H. (1972). Acidity
Measurements at Elevated
Temperatures: VI. Boric Acid Equilibria. Inorg.
Chem..

[23] Harris P. C. (1993). Chemistry
and Rheology of Borate-Crosslinked Fluids at Temperatures to 300F. J. Pet. Technol..

[24] Pettignano A., Grijalvo S., Häring M., Eritja R., Tanchoux N., Quignard F., Díaz Díaz D. (2017). Boronic Acid-Modified
Alginate Enables Direct Formation of Injectable, Self-Healing and
Multi-Stimuli-Responsive Hydrogels. Chem. Commun..

[25] Bishop M., Shahid N., Yang J., Barron A. R. (2004). Determination of
the Mode and Efficacy of the Cross-Linking of Guar by Borate Using
MAS 11B NMR of Borate Cross-Linked Guar in Combination with Solution
11B NMR of Model Systems. J. Chem. Soc., Dalton
Trans..

[26] Hurmaus T., Plank J. (2016). Behavior of titania
nanoparticles in cross-linking hydroxypropyl
guar used in hydraulic fracturing fluids for oil recovery. Energy Fuels.

[27] Belcher R., Tully G. W., Svehla G. (1970). A Comparative
Study of Various Complexing
Agents (Polyols) Used in the Titration of Boric Acid. Anal. Chim. Acta.

[28] Legemah M., Guerin M., Sun H., Qu Q. (2014). Novel High-Efficiency
Boron Crosslinkers for Low-Polymer-Loading Fracturing Fluids. SPE J..

[29] Guner P. ., Askun T., Er A. (2025). Antimicrobial potential of boron-containing
compounds: antibacterial, antifungal, and antimycobacterial activities. Byron J..

[30] Song S., Gao P., Sun L., Kang D., Kongsted J., Poongavanam V., Zhan P., Liu X. (2021). Recent developments in the medicinal
chemistry of single boron atom-containing compounds. Acta Pharm. Sin. B.

[31] Celebi O., Celebi D., Baser S. (2024). Antibacterial
Activity
of Boron Compounds Against Biofilm-Forming Pathogens. Biol. Trace Elem. Res..

[32] Syvolos Y., Salama O. E., Gerstein A. C. (2024). Constraint
on boric acid resistance
and tolerance evolvability in Candida albicans. Can. J. Microbiol..

[33] Iyigundoğdu Z. (2023). Synergistic
effects of zinc borate and graphene on enhanced thermal stability
and antimicrobial properties of poly­(methyl methacrylate). Polym. Compos..

[34] Silhavy T. J., Kahne D., Walker S. (2010). The bacterial cell envelope. Cold Spring Harbor Perspect. Biol..

[35] Seong M., Kondaveeti S., Choi G., Kim S., Kim J., Kang M., Jeong H. E. (2023). 3D Printable Self-Adhesive and Self-Healing
Ionotronic Hydrogels for Wearable Healthcare Devices. ACS Appl. Mater. Interfaces.

[36] Weizman O., Dotan A., Nir Y., Ophir A. (2017). Modified Whey
Protein
Coatings for Improved Gas Barrier Properties of Biodegradable Films. Polym. Adv. Technol..

[37] Ribeiro, T. T. B. ; Nunes, T. P. ; Jesus, B. G. ; Dariva, C. ; Padilha, F. F. ; Franceschi, E. ; Composição Filmogênica Antifúngica para Revestimento de Frutas. BR 102018000947–8 B1, 2022.

[38] Idahagbon N. B., Nicholas R. J., Wei A. (2025). Pectin–Cellulose Nanofiber
Composites: Biodegradable Materials for Modified Atmosphere Packaging. Food Hydrocolloids.

[39] Renard, C. M. G. C. ; Thibault, J.-F. Pectins in mild alkaline conditions: β-elimination and kinetics of demethylation. In Pectins and pectinases; Elsevier Science B.V.: Amsterdam, 1996; 603–608.

[40] Krall S. M., McFeeters R. F. (1998). Pectin
hydrolysis: effect of temperature, degree of
methylation, pH, and calcium on hydrolysis rates. J. Agric. Food Chem..

[41] Canteri M. H. G., Moreno L., Wosiacki G., Scheer A. de P. (2012). Pectina: da matéria-prima
ao produto final. Polímeros.

[42] Saito K., Xu T., Ishikita H. (2022). Correlation
between C = O stretching vibrational frequency
and pKa shift of carboxylic acids. J. Phys.
Chem. B.

[43] Manrique G. D., Lajolo F. M. (2002). FT-IR spectroscopy as a tool for measuring degree of
methyl esterification in pectins isolated from ripening papaya fruit. Postharvest Biol. Technol..

[44] Sanchez-Valle C., Reynard B., Daniel I., Lécuyer C., Martinez I., Chervin J. C. (2005). Boron isotopic fractionation between
minerals and fluids: New insights from in situ high pressure-high
temperature vibrational spectroscopic data. Geochim. Cosmochim. Acta.

[45] Pye, C. C. An Ab Initio Study of Boric Acid, Borate, and their Interconversion. In Progress in Theoretical Chemistry and Physics; Springer: Cham, 2018; 143–177. 10.1007/978-3-319-74582-4_8.

[46] Chen X., Ji N., Li F., Qin Y., Wang Y., Xiong L., Sun Q. (2022). Dual Cross-Linked Starch–Borax
Double Network Hydrogels with
Tough and Self-Healing Properties. Foods.

[47] Lv Y. K., Pan Z., Song C. Z., Chen Y. L., Qian X. (2019). Locust Bean Gum/Gellan
Gum Double-Network Hydrogels with Superior Self-Healing and pH-Driven
Shape-Memory Properties. Soft Matter.

[48] Thombare N., Jha U., Mishra S., Siddiqui M. Z. (2017). Borax Cross-Linked Guar Gum Hydrogels
as Potential Adsorbents for Water Purification. Carbohydr. Polym..

[49] Lim M., Kwon H., Kim D., Seo J., Han H., Khan S. B. (2015). Highly-Enhanced Water Resistant and
Oxygen Barrier
Properties of Cross-Linked Poly­(Vinyl Alcohol) Hybrid Films for Packaging
Applications. Prog. Org. Coat..

[50] Han J., Yue Y., Wu Q. (2017). Effects of nanocellulose on the structure and
properties of poly­(vinyl alcohol)-borax hybrid foams. Cellulose.

[51] Ge W., Cao S., Yang Y., Rojas O. J. (2019). Rapid Self-Healing, Stretchable,
Moldable, Antioxidant and Antibacterial Tannic Acid-Cellulose Nanofibril
Composite Hydrogels. Carbohydr. Polym..

[52] Tanpichai S., Phoothong F., Boonmahitthisud A. (2022). Superabsorbent
Cellulose-Based Hydrogels
Cross-Linked with Borax. Sci. Rep..

[53] Biswas A., Ghosh T., Gavel P. K., Das A. K. (2020). PEG functionalized
stimuli responsive self-healable injectable dynamic imino-boronate
G-quadruplex hydrogel for the delivery of doxorubicin. ACS Appl. Bio Mater..

[54] Sousa V., Amaral A. J. R., Castanheira E. J., Marques I., Rodrigues J. M. M., Félix V., Borges J., Mano J. F. (2023). Self-supporting
hyaluronic acid-functionalized G-quadruplex-based perfusable multicomponent
hydrogels embedded in photo-cross-linkable matrices for bioapplications. Biomacromolecules.

[55] Nakamura Y., Numata K., Hirosaki M., Miyajima H., Fujita S. (2026). Dynamic glycan
network engineering of native mucin enables reversible, self-healing,
and adhesive hydrogel interfaces. Nanoscale
Adv..

[56] Dave K., Nath H. K. (2018). Synthesis, Characterization
and Application of Disodium
Tetraborate CrossLinked Polyvinyl Alcohol Membranes for Pervaporation
Dehydration of Ethylene Glycol. Acta Chim. Slov..

[57] Verni E., Sabatini F., Lee C., Fiocco G., Weththimuni M. L., Vigani B., Lange H., Malagodi M., Volpi F. (2025). Development
and Characterization of Novel Tannin-Modified Konjac Glucomannan Hydrogels
with Optimized Crosslinking Features. Carbohydr.
Polym. Technol. Appl..

[58] Uyanga K. A., Daoud W. A. (2021). Green and Sustainable
Carboxymethyl Cellulose–Chitosan
Composite Hydrogels: Effect of Crosslinker on Microstructure. Cellulose.

[59] Pinto, E. N. M. G. Aditivação de Pastas Geopoliméricas com Tetraborato de Sódio e Látex Não Iônico para Cimentação de Poços de Petróleo, Master’s Thesis; Universidade Federal do Rio Grande do Norte: Natal, Brazil, 2007.

[60] Uddin K. M. A., Ago M., Rojas O. J. (2017). Hybrid
films of chitosan, cellulose
nanofibrils and boric acid: flame retardancy, optical and thermo-mechanical
properties. Carbohydr. Polym..

[61] Nguyen T. T. T., Ho H. T., Hoang D., Nguyen Q. A. P., Tran T. V. (2024). Novel Films
of Pectin Extracted from Ambarella Fruit Peel and Jackfruit Seed Slimy
Sheath: Effect of Ionic Crosslinking on the Properties of Pectin Film. Carbohydr. Polym..

[62] Zhang S., Pan X., Zhao J., Li J., Yu X., Peng Y., Wu J. (2023). Characterization of
Ionically Crosslinked Mango Peel Pectin-Based
Films: Effect of Different Cations on the Improved Properties of Film. Food Packag. Shelf Life.

[63] Coer O. E., Davidson B. L., Boardman B. M., Peters G. M. (2025). Modulating thermal
stability and flexibility in chitosan films with neutral polyol-boric
acid complexes. Biomacromolecules.

[64] Wenzel R. N. (1936). Resistance
of Solid Surfaces to Wetting by Water. Ind.
Eng. Chem..

[65] Deng Y., Peng C., Dai M., Lin D., Ali I., Alhewairini S. S., Zheng X., Chen G., Li J., Naz I. (2020). Recent development of super-wettable materials and
their applications
in oil–water separation. J. Cleaner Prod..

[66] Aitboulahsen M., Chairi H., Laglaoui A. (2020). Gelatin/pectin-based
film incorporated with essential oils: Functional characteristics
and shelf life extension of tilapia fillets under refrigeration. J. Food Saf..

[67] Oyekanmi A. A., Abdul Khalil H. P. S., Rahman A. A., Mistar E. M., Olaiya N. G., Alfatah T., Yahya E. B., Mariana M., Hazwan C. M., Abdullah C. K. (2021). Extracted
Supercritical CO_2_ Cinnamon Oil
Functional Properties Enhancement in Cellulose Nanofibre Reinforced
Eucheuma cottonii Biopolymer Films. J. Mater.
Res. Technol..

[68] Khalil R. K. S., Sharaby M. R., Abdelrahim D. S., ElLeithy A. E. (2024). A Novel Multifunctional
Carboxymethyl Cellulose Packaging Film with Encapsulated Lemon Oil
for Quality Enhancement of Cherry Tomato and Baby Spinach Leaves.
Food Packag. Food Packag. Shelf Life.

