# Supplementary material for: Borax as a Cross-Linking Agent in Pectin Films: Effects of Borax Concentration and Temperature on Film Properties
Source: ACS Omega. 2026 Jun 18;11(25):36667–78. doi: 10.1021/acsomega.5c12753 (PMC13325142; doi:10.1021/acsomega.5c12753)
Supplement: Supplementary file 1 [file ao5c12753_si_001.pdf]

Supporting Information

Borax as a crosslinking agent in pectin films: effects of borax concentration and temperature on film properties

Natalia V. G. Mendes[1]*; Luís F. Zitei-Baptista[2]; Fabricio B. Ferreira[3], Fabio R. M. Batista[1]; Delia R. Tapia-Blácido[2]; Eduardo R. Triboni[1*]

[1] Department of Chemical Engineering, Nanotechnology and Process Engineering Laboratory, Lorena School of Engineering, University of São Paulo, 12602-810, Lorena-SP, Brazil.

[2] Department of Chemistry, Laboratory of Agroindustrial Biopolymers, Ribeirão Preto Faculty of Philosophy, Sciences, and Letters, University of São Paulo, 14040-901, Ribeirão Preto-SP, Brazil.

[3] Department of Biotechnology, University of São Paulo, 12602-810, Lorena-SP, Brazil.

Corresponding Author:

*natalia_mendes@usp.br; *tribonier@usp.br

SI-1 Real-Time FTIR Spectroscopy

Real-time FTIR spectroscopy was carried out using a Mettler Toledo ReactIR 702L infrared probe. For data analysis the background spectra (solvents and solution components) were subtracted from the target sample spectra. Fig. SI-1 shows spectra of boric acid and borax in water, while Fig. SI-2 shows spectra of pectin in water at 50 °C overtime, and Fig. SI-3 shows boric acid in glycerol/water medium and in pectin/water medium.

By Fig. SI-1 we can identify stretches of the B–O acid boric bonds at 1408 cm^{-1} and 1144 cm^{-1}, while borax spectra (Fig. SI-1b) also showed peaks at 1320 cm^{-1} and at 960 cm^{-1}, 880 cm^{-1} and 856 cm^{-1}, which are attributed to B–O stretching in the $B(OH)_4^-$ moieties [1, 2]. Other studies have also assigned the region between 880 cm^{-1} to 830 cm^{-1} to the tetrahedral B-O stretches [3, 4]. Fig. SI-2 indicates that pectin does not undergo reaction or depolymerization at 50 °C. Fig. SI-3 shows stretches at 1408 cm^{-1} and 1140 cm^{-1} in all samples, which relates to the boric acid stretching (see Fig.S1a), showing no significant structural changes between glycerol/pectin and boric acid under acidic conditions.

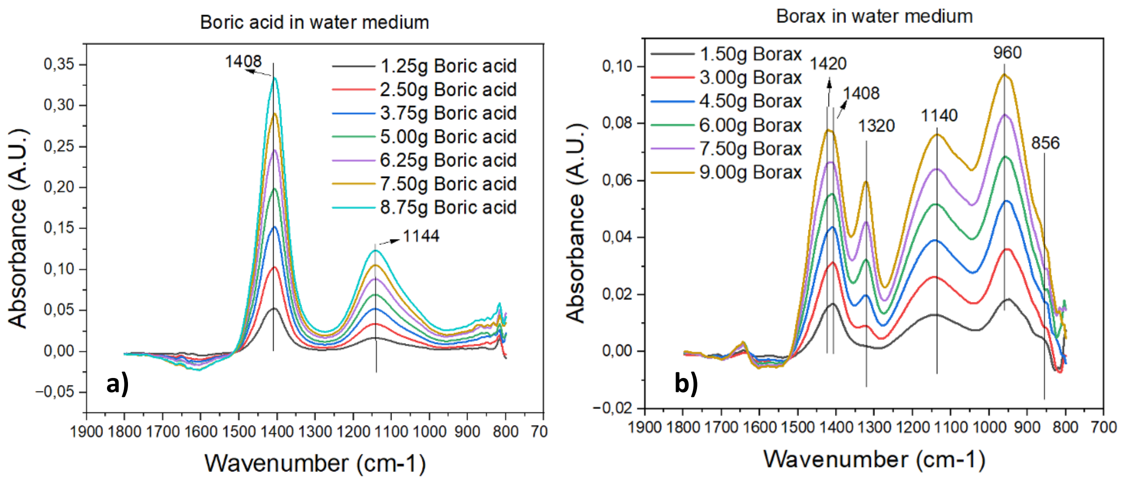

Fig. SI-1. In-Situ spectroscopy (infrared probe) in water (175mL) medium of: a) boric acid; b) borax.

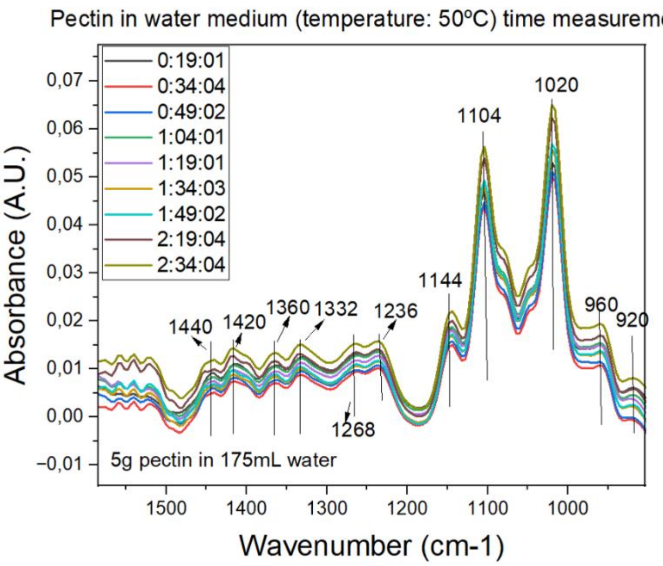

Fig. SI-2. In-Situ spectroscopy (infrared probe) of pectin (5g) in water (175mL) medium at temperature of 50ºC through time.

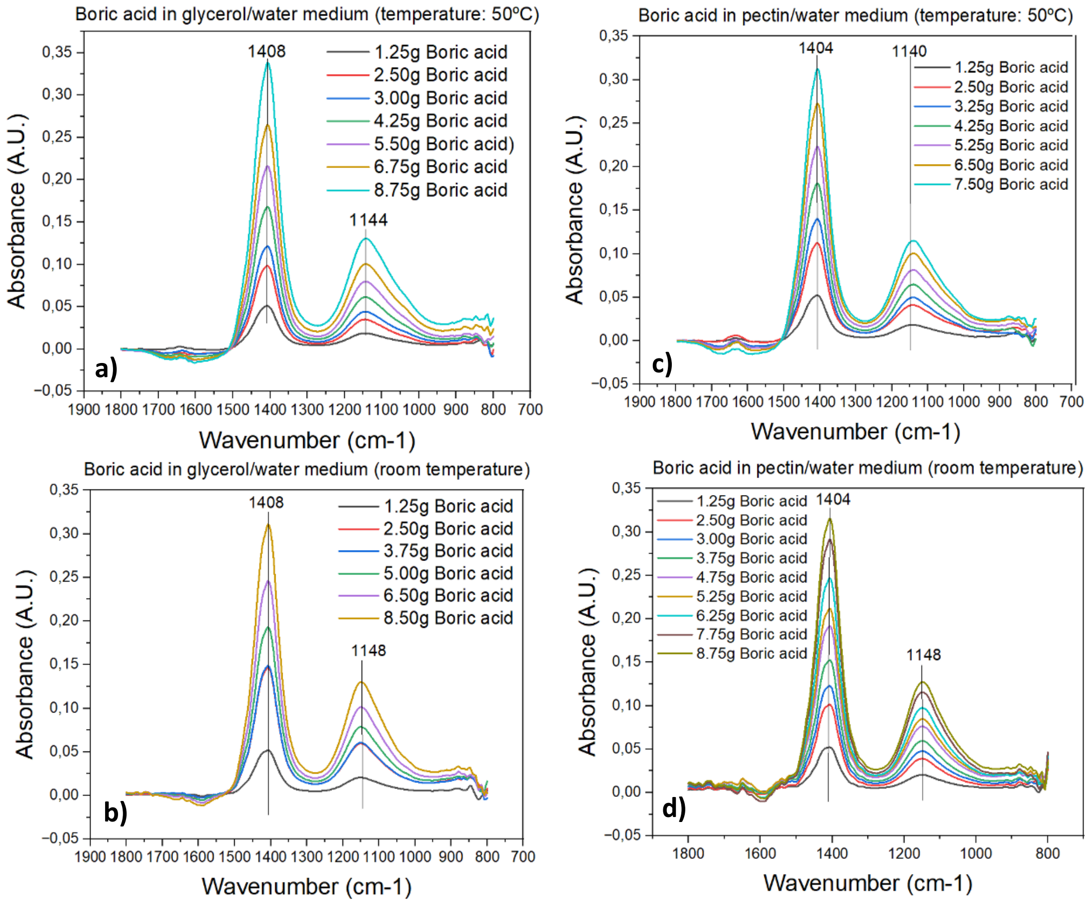

Fig. SI-3. In-Situ spectroscopy (infrared probe) of: a) boric acid in glycerol(5mL)/water(175mL) medium at temperature of 50ºC; b) boric acid in glycerol(5mL)/water(175mL) medium at room temperature; c) boric acid in pectin (5g)/water(175mL) medium at temperature of 50ºC; d) boric acid in pectin(5g)/water(175mL) room temperature.

SI-2 Possible mechanisms for B-O-C bonds formation

Pectin chain can undergo depolymerization (Fig. SI-4a) [5 – 7], mainly under high temperatures, which may contribute to pectin and boron species interactions/B-O-C bonds formation. Possible interactions between pectin OH groups and boron species are illustrated in Fig. SI-4b, c. However, a better interpretation of these interactions or bond formation will be achieved by ^{11}B-NMR in measurements and will be taken on further investigation.

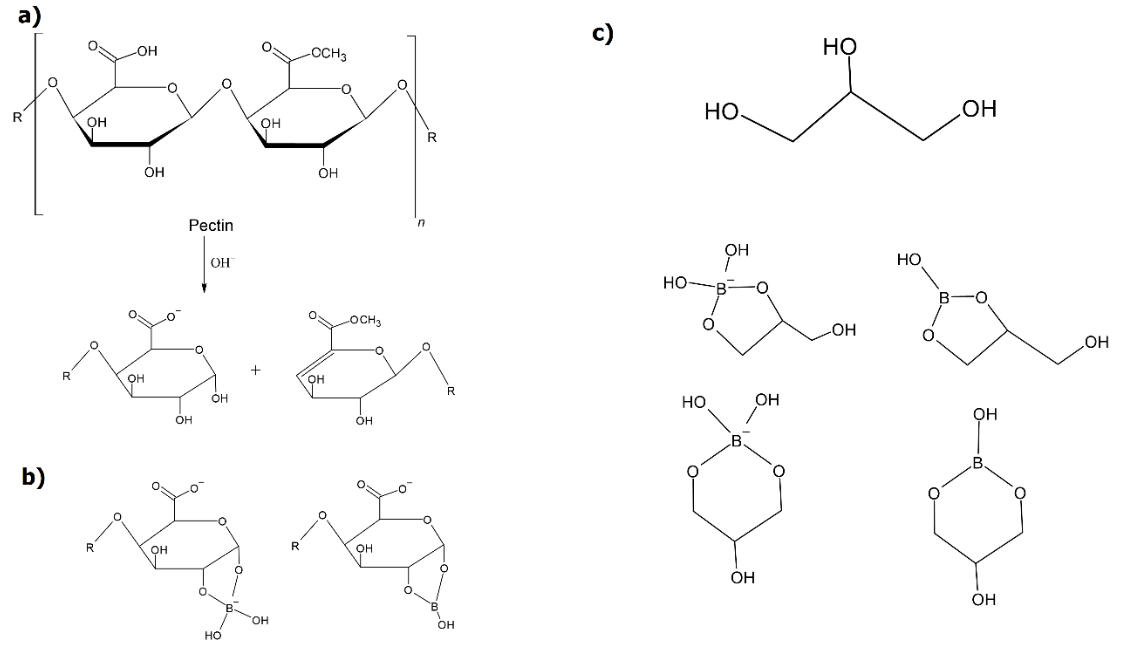

Fig. S4: Scheme representing: a) pectin depolymerization through β- elimination; b) trigonal and tetrahedral B-O-C bonds in pectin; c) glycerol and trigonal and tetrahedral B-O-C bonds. R denotes the continuation of the polymer (pectin) chain.

References:

1. Sanchez-Valle C, Reynard B, Daniel I, Lécuyer C, Martinez I, Chervin JC. Boron isotopic fractionation between minerals and fluids: New insights from in situ high pressure-high temperature vibrational spectroscopic data. Geochim Cosmochim Acta. 2005 Sep 1;69(17):4301-4313. doi:10.1016/j.gca.2005.03.054
2. Pye, C. C. An Ab Initio Study of Boric Acid, Borate, and their Interconversion. In *Progress in Theoretical Chemistry and Physics*, Springer: Cham, 2018; pp 143–177. DOI: 10.1007/978-3-319-74582-4_8.
3. Frost RL, Xi Y, Scholz R, Belotti FM, Filho MC. Infrared and Raman spectroscopic characterization of the borate mineral colemanite – $CaB_3O_4(OH)_3 \cdot H_2O$ – implications for the molecular structure. J Mol Struct. 2013;1057:23–28.

4. Huang, S.Q.; Su, S.Y.; Gan, H.B.; Wu, L.J.; Lin, C.H.; Xu, D.Y.; Zhou, H.F.; Lin, X.L.; Qin, Y.L. Facile fabrication and characterization of highly stretchable lignin-based hydroxyethyl cellulose self-healing hydrogel. Carbohydr. Polym. 2019, 223, 115080.
5. Renard, C. M. G. C.; Thibault, J.-F. Pectins in mild alkaline conditions: β-elimination and kinetics of demethylation. *Pectins and pectinases*. Amsterdam: Elsevier Science B.V., 1996. p. 603–608.
6. Krall SM, McFeeters RF. Pectin hydrolysis: effect of temperature, degree of methylation, pH, and calcium on hydrolysis rates. *J Agric Food Chem.* 1998;46(4):1311–1315.
7. Canteri MHG, Moreno L, Wosiacki G, Scheer A de P. Pectina: da matéria-prima ao produto final. Polímeros [Internet]. 2012;22(2):149–57. Available from: https://doi.org/10.1590/S0104-14282012005000024
